# Shielding Performance of Electromagnetic Shielding Fabric Implanted with “Split-Ring Resonator”

**DOI:** 10.3390/polym15061366

**Published:** 2023-03-09

**Authors:** Zhe Liu, Jin Duan, Xiuchen Wang

**Affiliations:** 1 School of Textile Science and Engineering, Xi’an Polytechnic University, Xi’an 710048, China; 2 Key Laboratory of Functional Textile Material and Product, Xi’an Polytechnic University, Ministry of Education, Xi’an 710048, China; 3 School of Apparel and Art Design, Xi’an Polytechnic University, Xi’an 710048, China

**Keywords:** split-ring resonator, implantation, electromagnetic shielding fabric, shielding effectiveness, improvement

## Abstract

The electromagnetic shielding (EMS) fabric is an important electromagnetic protection product, which is widely applied in various fields. The improvement of its shielding effectiveness (SE) has always been the focus of research. This article proposes to implant a metamaterial structure of a “split-ring resonator (SRR)” in the EMS fabrics, so that the fabric not only maintains the porous and lightweight characteristics, but also obtains the SE improvement. With the help of the invisible embroidery technology, stainless-steel filaments were used to implant hexagonal SRRs inside the fabric. The effectiveness and influencing factors of the SRR implantation were described by testing the SE of the fabric and analyzing the experimental results. It was concluded that the SRR implantation inside the fabric can effectively improve the SE of the fabric. For the stainless-steel EMS fabric, the increase amplitude of the SE reached between 6 dB and 15 dB in most frequency bands. The overall SE of the fabric showed a decrease trend with the reduction of the outer diameter of the SRR. The decrease trend was sometimes fast and sometimes slow. The decreasing amplitudes were different in various frequency ranges. The number of embroidery threads had a certain effect on the SE of the fabric. When other parameters remained unchanged, the increase of the diameter of the embroidery thread resulted in the increase of the SE of the fabric. However, the overall improvement was not significant. Finally, this article also points out that other influencing factors of the SRR need to be explored, and the failure phenomenon may occur under certain situations. The proposed method has the advantages of the simple process, convenient design, no pore formation, SE improvement retaining the original porous characteristics of the fabric. This paper provides a new idea for the design, production, and development of new EMS fabrics.

## 1. Introduction

Electromagnetic wave radiation can cause great electromagnetic damage to human body in many frequency bands, and it also causes serious interference to other electronic equipment around. In many cases, measures must be taken to shield its harm and impact [1]. The electromagnetic shielding (EMS) fabric is composed of synthetic polymers, natural polymers, and metal materials and is a flexible, lightweight, and low-cost electromagnetic wave protection material. It plays an important role in terms of electromagnetic wave shielding, and its demand is increasing. Developing EMS fabric products with excellent shielding effectiveness (SE) has become a hot topic of research in the electromagnetic protection field [2].

The primary methods of manufacturing EMS fabrics are blending and coating. Both methods have certain limitations. The SE is often low using the blending method due to the existence of pores in the blended fabric. The SE is improved via the coating method, but the fabric loses original comfort properties such as air permeability and moisture permeability because of pores plugging, and the weight of the fabric increases [3]. Therefore, the research of maintaining original properties and affording excellent shielding performance of the EMS fabric becomes an urgent need in this field.

However, the research of this aspect is still slow, and the existing mainly focuses on the numerical model calculation [4], structural characteristics [5], performance improvement [6,7], influencing factors analysis [8,9], test evaluation [10], new product development [11,12], and new mechanism research [13,14]. At present, improving the SE of the EMS fabric and retaining the inherent porous characteristics lack effective methods.

Therefore, this article proposes to implant a “split-ring resonator” metamaterial structure in the fabric, so that the SE can be improved based on retaining the pores of the fabric. The split-ring resonator (SRR) is a magnetic metamaterial structure. This term was first created by Hardy [15]. The scientist Pendry and the coworkers transformed its structure into the form of a concentric and reverse split ring [16]. The SRR has resonance characteristics. There is a narrow stopband near its resonance characteristics, which can effectively prevent the passage of electromagnetic waves and achieve the effect of shielding electromagnetic waves [17].

Over the years, fully manipulating the characteristics of electromagnetic waves has become the central goal of various modern technologies [18]. Using SRR has been an effective way to achieve this goal, and many scholars have conducted a lot of frontier exploration work. For example, the concept of the optical bound state in the continuum (BIC) was introduced into the design of a structured metal element surface to enhance the light-matter interaction in nano-optics and quantum photonics, thus enhancing the resonance field in plasmonic metasurfaces [19]. By judiciously designing two-dimensional THz metamaterials consisting of a resonant metallic structure embedded in the dielectric environment, the electromagnetic field of the incident THz pulse was enhanced to have values sufficiently high to cause nonlinear responses of the environment [20]. A reconfigurable hybrid metasurface platform was proposed by incorporating the phase-change material Ge2Sb2Te5 (GST) into metal-dielectric meta-atoms for active and nonvolatile tuning of properties of light [21]. These studies have greatly promoted the development and practical application of the SRR theory.

At present, the application of the SRR in the field of EMS fabric has been rarely reported. The existing relevant literature mainly focuses on the research of textile-based antennas [22,23], sensors [24,25], and filtering [26,27], which mention their SE and absorbing bandwidths and other characteristics. For example, in order to filter and control the signal propagation along the ultra-high frequency (UHF) range of e-textile, transmission lines with one or more SRRs were loaded on a felt substrate, and a conclusion was obtained that the stopband level of metamaterial electronic textiles with compact embroidery was high. Hossain et al. [28] introduced a 10-corner C-shaped complementary split-ring resonator (CSRR) textile-based metamaterial (MTM) and pointed out that the satisfactory consistency between the simulation and the experiment of the material, which indicated the potential of the proposed MTM in microwave applications. Gil et al. [29] designed, simulated and tested an embroidered metamaterial monopole antenna based on the electromagnetic bandgap shielding structure of an SRR and studied the influence of different types of embroidered metamaterial patterns on the antenna performance, shielding effect, and personal safety.

The SRR has been also widely used in other electromagnetic fields due to its excellent performance. For example, Yarici et al. [30] proposed an experimental design method based on the study of the influence of the geometric parameters of the inverted square SRR on its resonant frequency, which provided a new path for microwave research. Xu et al. [31] put forward a design idea of an SRR metamaterial with a tunable terahertz free spectral range (FSR), which could be widely used in double/four resonant switches, polarization switches, and efficient environmental sensors. Sahu et al. [32] made and measured a metamaterial polarization converter of four resonant single band using a non-destructive free-space microwave measurement device. The results showed that the method has great potential in various practical electromagnetic applications. Although these methods cannot be used in EMS fabrics, they have positive reference value for the research of this article due to their theoretical analysis and practical verification of the SRR.

This article proposes for the first time to use the invisible embroidery technology to implant an SRR inside the fabric. By taking advantage of its loss characteristics of the electromagnetic wave, the shielding performance of the fabric was improved in the frequency band of 6.57 GHz–9.99 GHz. The effectiveness of the SRR and the influence of the main parameters on the SE of the fabric were analyzed. This method offers a new idea for improving the SE of the EMS fabrics and provides a reference for the design and manufacture of lightweight EMS fabrics.

## 2. Experiment

### 2.1. Basic Ideas

**Figure 1:**
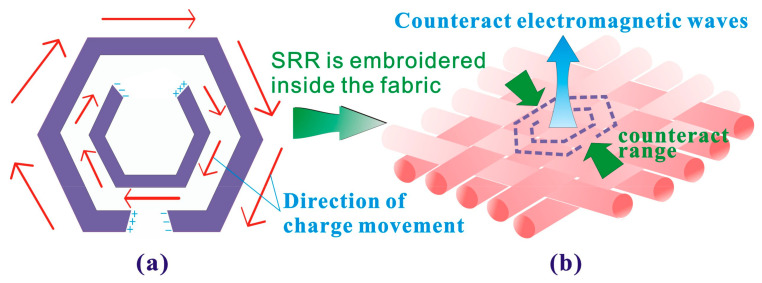
SRR implantation for the improvement of the SE of the EMS fabric. (**a**) Split-ring resonator (SRR) and (**b**) Functional embroidery.

The SRR is a metamaterial structure, which is composed of a pair of concentric split rings with a subwavelength size. It can effectively change the magnetic permeability, producing good loss effects on electromagnetic waves [33]. As shown in Figure 1a, in this structure, an opening is added on a conductive ring to make it equivalent to a resonant circuit composed of inductance and capacitance. Two concentric split rings with different sizes are placed in reverse, which can offset the electric dipole moments generated by the two rings, thereby enhancing the electromagnetic wave loss effect of the SRR [34]. As shown in Figure 1b, this article selects conductive stainless-steel filaments and the invisible embroidery technology to form SRRs with different parameters in the EMS fabric, use the SRR characteristics to counteract electromagnetic waves and achieves the purpose of improving the SE of the EMS fabric.

### 2.2. Implantation Method of the SRR

**Figure 2:**
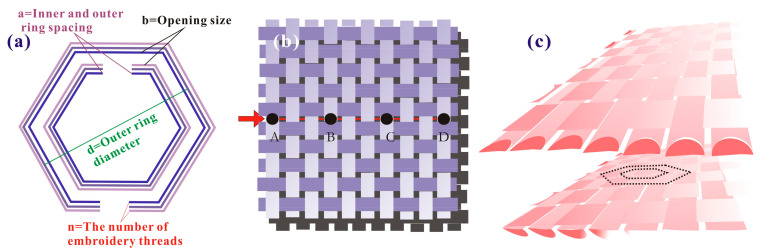
SRR model and invisible embroidery method. (**a**) SRR model, (**b**) Invisible functional embroidery trajectory, (**c**) Pattern position of invisible functional embroidery.

This article explores the construction of a hexagonal SRR model and implants it into the fabric, as shown in Figure 2a. The model parameters include the inner and the outer ring spacing a, the opening size b, the outer ring diameter d, and the number of embroidery threads n. According to the experimental summary and the electromagnetic theory analysis, each parameter should meet the following requirements:

(1) $$\frac{1}{10}<\frac{a}{d}<\frac{3}{10}$$

(2) $$\frac{2\pi d}{10}<b<\frac{6\pi d}{10}$$

The implantation process is completed by manual embroidery operation. According to the needs of the experiments, SRRs with different outer diameters and different threads are implanted into the fabric. To prevent conventional embroidery from producing a large number of pinholes that can reduce the SE, an invisible embroidery method is invented, as shown in Figure 2b. Assuming there are four embroidery points, i.e., A, B, C, and D, on the fabric, the embroidery needle picks up the surface of the fabric fiber at point A, passes through the middle of the fiber, passes through points B and C in turn and finally passes out of point D to complete the SRR implantation process. The needle always walks inside the fabric with this method, which will not cause damage to the fabric structure. This is equivalent to placing a complete pattern among the fibers, as shown in Figure 2c.

### 2.3. Test Equipment

**Figure 3:**
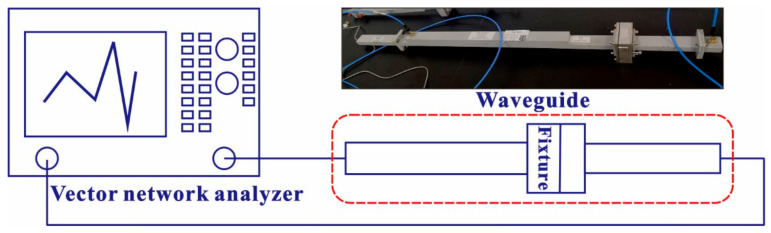
Test equipment of the SE.

As shown in Figure 3, the SE of the EMS fabric was tested with a vector network analyzer (Agilent Technology Co., Ltd., Santa Clara, CA, USA, N5232A) and a waveguide (Xi’an Hengda Microwave Technology Development Company, Xi’an, China, BJ84). The frequency band was 6.57 GHz–9.99 GHz, and the size of the fabric sample was 4.5 mm × 3.2 mm. By reading the S parameter of the network analyzer, the SE of the fabric can be calculated as:

(3) $$\mathrm{SE}==10\mathrm{lg}{\left(S11\right)}^{2}$$

where S11 is the input reflection coefficient, which represents the amount of electromagnetic waves reflected in the medium. It is an important parameter for calculating the electromagnetic shielding performance. The smaller the value of S11, the better the electromagnetic shielding performance of the fabric. In order to reduce errors, the test results were taken as the average of five tests.

### 2.4. Experimental Materials

EMS fabrics containing 30% stainless steel fibers were selected as base cloth 1, and ordinary fabrics containing 100% cotton fibers were selected as base cloth 2. Their parameters are listed in Table 1. The diameter of the embroidery needle was 0.1 mm, and the embroidery threads were formed twisting the conductive stainless-steel filaments with a diameter of 4 um.

## 3. Results and Analysis

### 3.1. Effectiveness of the SRR Implantation

**Figure 4:**
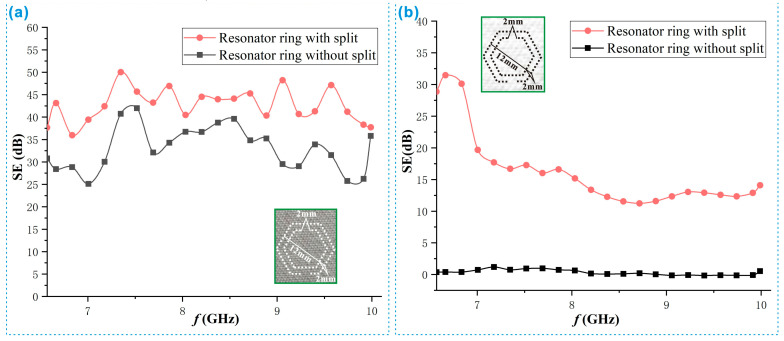
Comparison of the SE of the fabric before and after the SRR implantation. (**a**) Stainless steel EMS fabric and (**b**) Ordinary cotton fabric.

The experiments proved that implanting SRRs into the fabric, whether it was EMS fabric or ordinary fabric, could improve the SE of the fabric, which have fully proved the effectiveness of the SRR for the SE enhancement. Figure 4 shows the SE comparison before and after the SRR was implanted in the base cloth 1 and the base cloth 2. The outer ring diameter of the SRR was 1.2 cm. Considering the width of the test sample, the number of SRRs was 2. The spacing between the inner and outer rings a was 1 mm. The opening size b was 2 mm. Figure 4a shows that the SE of the fabric was significantly improved in all frequency bands after implanting the SRR into the original stainless-steel fabric with shielding performance. In most frequency ranges, the increase reached the ranges between 6 dB and 15 dB. It illustrates that the effect of the SRR was obvious and the SE of the stainless-steel fabric was significantly enhanced after being implanted with the SRR. Figure 4b shows that the SE of the cotton fabric was also significantly improved after the SRR was implanted. The improvement was from the initial unshielded effect to the SE with the maximum value of about 32 dB and the minimum value of about 11 dB. At the same time, the shielding effect first increased and then decreased with the frequency increase, and the trend was stable when the frequency was higher than 8.5 GHz.

**Figure 5:**
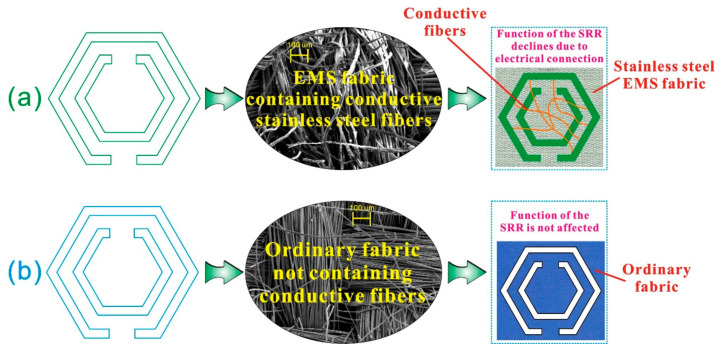
Improving mechanisms of the SE of the SRR in different fabrics. (**a**) Mechanism explanation for Figure 4a; (**b**) Mechanism explanation for Figure 4b.

The reason for the above phenomenon is related to the interference of the SRR in different fabrics. Figure 5 shows the mechanism explanation of the phenomenon in Figure 4, in which the electron micrograph was taken by a field-emission scanning electron microscope (MERLIN Compact, Carl Zeiss, Germany). As mentioned above, the inner and outer rings of the SRR need to be constructed with conductive media, and there is no communication between the inner and outer rings [35]. When the SRR was in the stainless-steel EMS fabric as shown in Figure 5a, there might be local fine electrical connections between the inner and outer rings of the SRR and in the opening area due to the presence of a large number of conductive stainless-steel fibers. To a certain extent, the strength of inductance and capacitance was weakened and therefore the shielding effect of the SRR was suppressed. However, this suppression was weak, and the function of the SRR was not lost significantly. Therefore, it still dramatically improved the SE of the EMS fabric. It was this limited weakening that made the effect of the SRR not very obvious with the change of the frequency. As a result, the SE of the EMS fabric was improved evenly in all frequency bands. When the SRR was in the cotton fabric as shown in Figure 5b, the inner and outer rings and openings could not form electrical connection because of no containing conductive fibers. The function of the SRR was normal and it could play a great role, and therefore the SE of the fabric was improved uniformly in all frequency bands. For the phenomenon in Figure 4b that the SE of cotton fabric has improved greatly at frequencies lower than 7.1 GHz, it was determined by the properties of the SRR. That is, the stopband formed in the frequency range of 6.57 GHz–7.1 GHz can more effectively prevent the passage of the electromagnetic waves. Obviously, this effect was weakened due to the presence of the conductive fibers in Figure 4b.

### 3.2. Influence of the Size of the SRR on the SE

**Figure 6:**
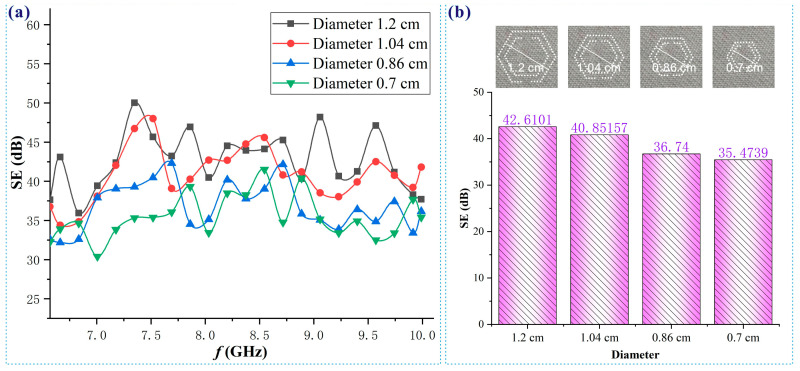
SE values of the fabrics with different diameters of the SRR. (**a**) Variation of the SE and (**b**) Average value of the SE.

The experiments showed that the size of the SRR also had an obvious effect on the SE of the fabric. Figure 6a is a comparison of the SE of the EMS cloth 1 when the outer ring diameters of the SRR were 1.2 cm, 1.04 cm, 0.86 cm, and 0.7 cm. It was observed that the SE of the fabric decreased with the reduction of the outer ring diameter. However, for different frequency ranges, the declining amplitude was not consistent. For example, when the diameter changed from 1.2 cm to 0.86 cm, the SE decreased by 12 dB near the frequency of 7.3 GHz, while it only decreased by 1 dB near the frequency of 7.7 GHz. From the perspective of the descending speeds, they were slow when the diameter decreased from 1.2 cm to 1.04 cm, and they were even close in many frequency bands. When the diameter decreased to 0.86 cm, the SE value decreased quickly. When the diameter continued to change to 0.7 cm, the descending speed slowed down again, resulting in closer SE values between the diameters of 0.86 cm and 0.7 cm. This phenomenon illustrated that the SE usually decreased in the sometimes fast and sometimes slow manner as the diameter decreased.

Figure 6b shows the average value of the SE with different outer ring diameters, which further confirmed the rule in Figure 6a. Specifically, the overall SE of the fabric decreased, as the outer diameter of the SRR decreased. The reason for this phenomenon is that the spacing distance between the two rings increases with the reduction of the size of the SRR resulted in reducing the function area of the SRR, and thus average SE of the fabric decreased. However, this reduction was obviously not significant. From another point of view, it also showed its function did not decrease significantly when the diameter of the SRR decreased but still maintained the function similar to that of the original size. In this way, the SRR still played a great role in the case of the increased spacing distance and did not appreciably reduce the SE of the fabric.

### 3.3. Influence of the Number of Embroidery Threads of the SRR Implantation on the SE

**Figure 7:**
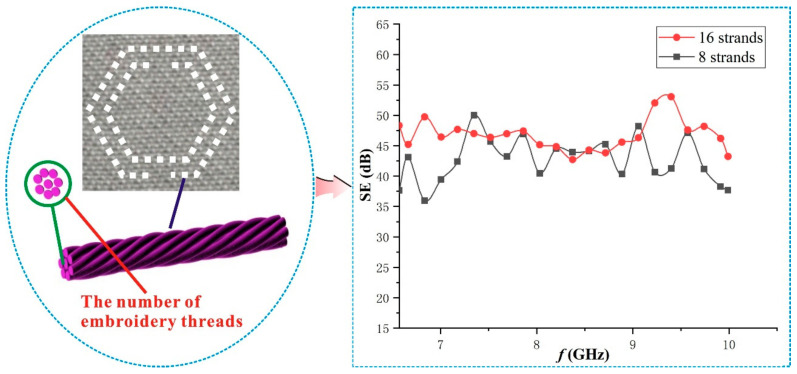
Influence of embroidery threads on the SE of the fabric.

It was observed from experiments that the number of embroidery threads had a certain effect on the SE of the fabric. Figure 7 shows the SE of the fabrics with 8 strands and 16 strands embroidery threads. It can be seen that the SE of the fabric increased with the increase of the number of the embroidery threads when the other parameters were unchanged, but the overall improvement was not significant. In the frequency bands between 6.57 GHz and 7.4 GHz, the SE of the fabric with 16 strands was greater than that with 8 strands. The SE of the fabric with 16 strands was significantly higher than that of the fabric with 8 strands in the frequency bands between 9.1 GHz and 9.6 GHz. The SE values of the fabric with 16 strands and 8 strands were close to each other in most other frequency bands, indicating that the change of the number of the embroidery threads has little effect on the overall SE.

The reason for the above phenomenon is that the role of the SRR is mainly related to the conductivity. In the general frequency bands of the electromagnetic wave, there is little difference in conductivity among 16 strands, 8 strands or even other strands. The strength of the inductance and capacitance generated by the SRR does not decrease significantly with the decrease of the number of embroidery threads, so that the number of the embroidery threads has little impact on the SE of the fabric.

From the above-mentioned, although the SE of the fabric was improved with the increase of the number of embroidery threads, the overall extent of the improvement was not obvious. At this time, the increase of the number of embroidery threads promoted the difficulty of embroidery and affected the beauty of the fabric. Similarly, although the SE of the fabric was not significantly reduced if the number of embroidery threads was too small, it was also difficult to embroider because it was too thin and the pattern was easy to deform or even break. Therefore, the number of embroidery threads must be moderate for embroidery. In constant experiments, it was found that the number of embroidery threads was eight strands, which is an appropriate parameter.

### 3.4. Other Influencing Factors and Invalidity

**Figure 8:**
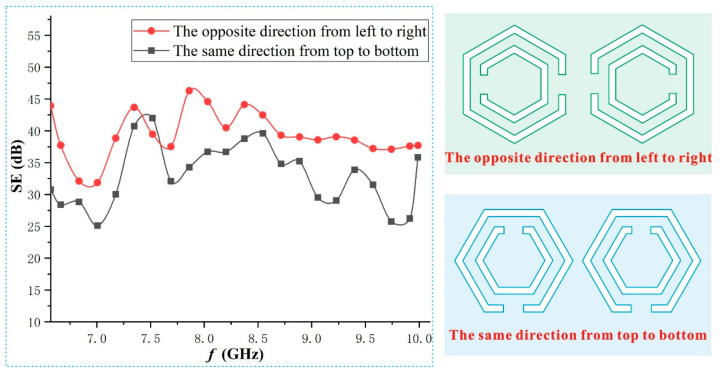
Influence of the SRR arrangement on the SE of the fabric.

From the above analysis, the unique advantages of embroidering the SRR into the fabric are the simple process, convenient design, and no pores and the improvement of the SE while maintaining the original characteristics of the fabric. Certainly, from experiments, it was found there are many influencing factors of the SRR on the SE of the fabric, such as the opening size, the arrangement spacing, the quantity per unit area, the parameter compatibility, the embroidery method, the fabric type, and the frequency band. For example, Figure 8 gives the influence of different arrangements of the same SRR on the SE. The SE of the fabric with the SRR opening direction in the opposite direction from left to right was greater than that of the fabric with the opening direction in the same direction from top to bottom. In fact, when the openings were arranged in other ways, the SE of the fabric also changed significantly. These proved that the arrangement of the openings has a great impact on the SE of the fabric. However, the cause of this phenomenon is still unclear. We preliminarily infer that it may be related to the direction combination of the inductance and capacitance generated by the SRR.

It can be seen that some of the mentioned influencing factors may have important effects on the SRR and the rules and mechanisms need to be clarified. However, it is a new attempt to implant SRRs in the fabric, and the changes of the involved parameters are more complex. At the same time, the size of the sample cloth measured via the experimental equipment is limited, the specific rules and mechanisms of the effect of these parameters on the SE are still unclear. We will continue to explore this aspect in the future.

In addition, we also found the invalidity of the SRR in the experiments. In some cases, the SRR could not achieve the desired purpose and had no obvious or even no effect on improvement of the SE of the fabric. At present, it is preliminarily believed that the reason for this phenomenon may be that the electrical connection formed by the SRR and the fabric is too dense, which seriously affects the formation of the inductance and the capacitance. These afford a functional failure. However, this is only a preliminary judgment. We believe that there must be other reasons, and we will continue to study this aspect in the future to clarify its formation conditions and mechanism.

## 4. Conclusions

(1) The method of invisible embroidery can be used to implant SRRs with different parameters in the fabric with conductive fibers. The method does not form piercing holes in the fabric and reduces the adverse effects of the embroidery needle on the improvement of the SE of the fabric.
(2) Implanting SRRs in the fabric could effectively improve the SE of the fabric. For the ordinary fabric, the SE was improved by about 32 dB at most and 11 dB at least. For the EMS fabric, the overall SE was significantly improved, and the improving range could reach between 6 dB and 15 dB.
(3) The pattern size of the SRR had a certain effect on the SE of the fabric. The overall SE of the fabric decreased with the decrease of the outer diameter of the SRR. The decrease trend was sometimes fast and sometimes slow. For different frequency ranges, the decreasing amplitude was not consistent.
(4) The number of embroidery threads of the SRR had a certain effect on the SE of the fabric. When other parameters remained unchanged, the increase of the number of embroidery threads increased the SE of the fabric, but the overall improvement was not significant.
(5) There are many influencing factors of the SRR on the SE of the fabric, such as the opening size, the arrangement spacing, the quantity per unit area, the parameter compatibility, the embroidery method, the fabric type, and the frequency band. At the same time, the SRR will also lose efficacy under certain circumstances.

Through the experiments and conclusions in this paper, we are convinced that the implantation of SRR is a new means to ensure the lightweight and comfortable performance of the EMS fabric and improve its SE. In addition, due to the variety of parameters such as the pattern, size, and arrangement of the SRR, the improvement of the electromagnetic performance of the fabric will certainly bring more surprises, which will be continuously explored and revealed in the follow-up work.

## Figures and Tables

**Table 1:** Parameters of fabric samples.

Fabric Name	Base Cloth 1—EMS Fabric	Base Cloth 2—Ordinary Fabric
Fiber content	30% stainless steel/40% polyester/30% cotton	100% cotton
Fabric weave	Plain	Plain
Warp density and weft density/(Number·10 cm^−1^)	148 × 129	298 × 139
Thickness (mm)	0.26	0.21
Yarn density (tex)	32	18.5

## Data Availability

Not applicable.

## References

[1] Mannan M., Weldu Y.W., Al-Ghamdi S.G. (2020). Health impact of energy use in buildings: Radiation propagation assessment in indoor environment. Energy Rep..

[2] Liu Z., He S., Wang H., Wang X. (2022). Improvement of the electromagnetic properties of blended electromagnetic shielding fabric of cotton/stainless steel/polyester based on multi-layer MXenes. Text. Res. J..

[3] Wang X., Hang G., Liu Z., He S. (2021). Study on finishing and electromagnetic properties of electromagnetic shielding fabric based on multilayer Ti3C2Tx medium. J. Text. Inst..

[4] Liu Z., Wang X. (2020). FDTD Numerical Calculation of Shielding Effectiveness of Electromagnetic Shielding Fabric Based on Warp and Weft Weave Points. Ieee Trans. Electromagn. Compat..

[5] Yin J., Ma W., Gao Z., Lei X., Jia C. (2022). A Structural Design Method of 3D Electromagnetic Wave-Absorbing Woven Fabrics. Polymers.

[6] Zhang X.L., Jin Z.M., Hu L.Z., Zhou X.Y., Yang K., Kremenakova D., Militky J. (2021). A Silver Yarn-Incorporated Song Brocade Fabric with Enhanced Electromagnetic Shielding. Materials.

[7] Pusic T., Saravanja B., Malaric K., Luburic M., Kaurin T. (2022). Electromagnetic Shielding Effectiveness of Woven Fabric with Integrated Conductive Threads after Washing with Liquid and Powder Detergents. Polymers.

[8] Wang X.C., Liu Z., Wu L., Wang Y., Su Y. (2021). Influencing factors and rules of shielding effectiveness of electromagnetic shielding clothing sleeve. Int. J. Cloth. Sci. Technol..

[9] Toghchi M.J., Loghin C., Cristian I., Campagne C., Bruniaux P., Cayla A., Lucano N., Chen Y. (2020). The effects of the structural parameters of three-dimensional warp interlock woven fabrics with silver-based hybrid yarns on electromagnetic shielding behavior. Text. Res. J..

[10] Vohra N., El-Shenawee M. (2021). K- and W-Band Free-Space Characterizations of Highly Conductive Radar Absorbing Materials. Ieee Trans. Instrum. Meas..

[11] Uzun S., Han M.K., Strobel C.J., Hantanasirisakul K., Goad A., Dion G., Gogotsi Y. (2021). Highly conductive and scalable Ti3C2Tx-coated fabrics for efficient electromagnetic interference shielding. Carbon.

[12] Tunakova V., Tunak M., Tesinova P., Seidlova M., Prochazka J. (2020). Fashion clothing with electromagnetic radiation protection: Aesthetic properties and performance. Text. Res. J..

[13] Yang Y.L., Wang J.P., Liu Z., Wang Z.J. (2021). A new study on the influencing factors and mechanism of shielding effectiveness of woven fabrics containing stainless steel fibers. J. Ind. Text..

[14] Kumar S., Kumar P., Gupta R., Verma V. (2021). Electromagnetic interference shielding behaviors of in-situ polymerized ferrite-polyaniline nano-composites and ferrite-polyaniline deposited fabrics in X-band frequency range. J. Alloy. Compd..

[15] Hardy W.N., Whitehead L.A. (1981). Split-Ring Resonator for Use in Magnetic-Resonance from 200-2000 mHz. Rev. Sci. Instrum..

[16] Pendry J.B., Holden A.J., Stewart W.J., Youngs I. (1996). Extremely low frequency plasmons in metallic mesostructures. Phys. Rev. Lett..

[17] Dey S., Chatterjee D., Garboczi E.J., Hassan A.M. (2020). Plasmonic Nanoantenna Optimization Using Characteristic Mode Analysis. Ieee Trans. Antennas Propag..

[18] Qiu C.-W., Zhang T., Hu G., Kivshar Y. (2021). Quo Vadis, Metasurfaces?. Nano Lett..

[19] Liang Y., Koshelev K., Zhang F., Lin H., Lin S., Wu J., Jia B., Kivshar Y. (2020). Bound States in the Continuum in Anisotropic Plasmonic Metasurfaces. Nano Lett..

[20] Kang B.J., Rohrbach D., Brunner F.D.J., Bagiante S., Sigg H., Feurer T. (2022). Ultrafast and Low-Threshold THz Mode Switching of Two-Dimensional Nonlinear Metamaterials. Nano Lett..

[21] Abdollahramezani S., Hemmatyar O., Taghinejad M., Taghinejad H., Kiarashinejad Y., Zandehshahvar M., Fan T.R., Deshmukh S., Eftekhar A.A., Cai W.S. (2021). Dynamic Hybrid Metasurfaces. Nano Lett..

[22] Shanmuganantham T., Kumar S.A., Sindhahaiselvi D. (2022). CB-CPW Fed SRR Loaded ISM and 5G Low Profile Antenna for On-Body Healthcare Monitor. Prog. Electromagn. Res. M.

[23] Agus A.N.S.S., Sabapathy T., Jusoh M., Abdelghany M.A., Hossain K., Padmanathan S., Al-Bawri S.S., Soh P.J. (2022). Combined RIS and EBG Surfaces Inspired Meta-Wearable Textile MIMO Antenna Using Viscose-Wool Felt. Polymers.

[24] Yang X., Tian X., Zeng Q., Li Z., Nguyen D.T., Ho J.S. (2022). Localized Surface Plasmons on Textiles for Non-Contact Vital Sign Sensing. Ieee Trans. Antennas Propag..

[25] Wiltshire B.D., Mirshahidi K., Nadaraja A.V., Shabanian S., Hajiraissi R., Zarifi M.H., Golovin K. (2021). Oleophobic textiles with embedded liquid and vapor hazard detection using differential planar microwave resonators. J. Hazard. Mater..

[26] Mashagba H.A., Rahim H.A., Adam I., Jamaluddin M.H., Yasin M.N.M., Jusoh M., Sabapathy T., Abdulmalek M., Al-Hadi A.A., Ismail A.M. (2021). A Hybrid Mutual Coupling Reduction Technique in a Dual-Band MIMO Textile Antenna for WBAN and 5G Applications. IEEE Access.

[27] Hossain K., Sabapathy T., Jusoh M., Soh P.J., Jamaluddin M.H., Al-Bawri S.S., Osman M.N., Ahmad R.B., Rahim H.A., Mohd Yasin M.N. (2021). Electrically Tunable Left-Handed Textile Metamaterial for Microwave Applications. Materials.

[28] Hossain K., Sabapathy T., Jusoh M., Soh P.J., Al-Bawri S.S., Osman M.N., Rahim H.A., Torrungrueng D., Akkaraekthalin P. (2022). Decagonal C-Shaped CSRR Textile-Based Metamaterial for Microwave Applications. Cmc-Comput. Mater. Contin..

[29] Gil I., Seager R., Fernandez-Garcia R. (2018). Embroidered Metamaterial Antenna for Optimized Performance on Wearable Applications. Phys. Status Solidi A-Appl. Mater. Sci..

[30] Yarici I., Ozturk Y. (2021). Analysis of an inverted square SRR via design of experiment (DoE) approach. J. Electr. Eng. -Elektrotechnicky Cas..

[31] Xu T., Xu X.C., Lin Y.S. (2021). Tunable Terahertz Free Spectra Range Using Electric Split-Ring Metamaterial. J. Microelectromechanical Syst..

[32] Sahu A., Chaudhary V., Yadav R., Panwar R. (2021). Design, Fabrication and Non-destructive Microwave Measurement of a Quad Resonance, Mono-band Metamaterial Polarization Converter Realized by a Fractal Inspired Split-Ring Resonator. J. Electron. Mater..

[33] Sebastian A., Joseph D., Aswathi P.V., Simon S.K., Bindu C., Joseph V.P., Andrews J. (2022). Complex permittivity measurement technique using metamaterial broadside coupled split ring resonator. J. Appl. Phys..

[34] Al-Naib I., Ateeq I.S. (2022). Excitation of Asymmetric Resonance with Symmetric Split-Ring Resonator. Materials.

[35] Bing S., Chawang K., Chiao J.C. (2022). A Self-Tuned Method for Impedance-Matching of Planar-Loop Resonators in Conformable Wearables. Electronics.

